# Miscellaneous Cases

**Published:** 1828-07

**Authors:** 


### MISCELLANEOUS CASES.

### *Case of open Foramen Ovale.*

James Spelman, æt. eighteen, was admitted into the Hardwicke Hospital, February 6, 1827, as a fever patient, under the care of Dr. Crampton. The thoracic viscera appeared to suffer from inflammation; he had a severe cough; complained of pain in different parts of his chest, chiefly at the left side; expectorated blood, and was distressed in his breathing. After two venesections and other remedies, on the 14th he appeared convalescent. From premature exposure, however, in a ward, which at this season was unusually cold, he soon suffered a relapse. The symptoms which now assailed him were those of acute rheumatism, with which almost all his joints were occupied, being excessively tumid, painful, and accompanied with a high degree of fever. In a few days the swellings of the joints abated, but the rheumatism became metastatic, the pectoral organs being again attacked, more especially the heart, in the region of which he appeared to suffer extremely. The pulse, during the first attack of fever, had been full and frequent, but regular: in the subsequent rheumatic attack, it preserved the same character; but, as the attending pyrexia appeared to subside, an occasional intermission or irregularity was observed.

It was necessary to adopt a very active practice. Repeated bleedings and other modes of relief were resorted to, otherwise it was obvious his respiratory organs would have been overpowered. It was equally clear that a cold fever ward was not a place suited

* Ibid.

to his case, which had now become complicated, but of a more chronic character. Indeed, the conclusion which Dr. C. was at this time disposed to adopt was, that severe organic lesion in the heart and lungs had occurred as the sequela of fever and acute rheumatism in a subject perhaps strongly predisposed to disorder in those important viscera.

On the 27th of February, the symptoms were—pain in the region of the heart, severe cough with a croupy sound, much muco-purulent expectoration, great distress in his breathing. At this time he stated, he had for years been occasionally subject to pain in the left side, but particularly after running, or any active exertion; his pulse extremely rapid, sometimes irregular; countenance pale, feet œdematous. The stethoscope indicated acute bronchitis, with hypertrophy, and disordered action in the heart, the motions of that organ being tumultuous and irregular.

On the 3d of March, the pulse became slower, about eighty, but very irregular.

On the 4th, he suffered extreme distress in his breathing,—or *smothering*, as he expressed it.

On the 5th, pulse again so rapid as not to be counted; he complained of great pain in the region of the heart. He was evidently growing worse.

On the 6th, a slight amelioration took place, but from this period until the 10th he gradually sunk. On this day he expired.

*Dissection.*—The thoracic cavity was examined on the 12th. On opening it, the lungs did not collapse, but the external appearance of this organ was healthy; the parenchymatous tissue exhibited some portions red, congested, and evidently inflamed; the bronchia vascula, and filled with a muco-purulent effusion.

Pericardium contained about two ounces and a half of fluid, in which a considerable quantity of coagulable lymph was seen; inner surface of this membrane not inflamed, but that portion of it which is reflected over the heart showed marks of inflammation in several places; both ventricles were much enlarged, exhibiting considerable hypertrophy; the semilunar aortic valves showed recent fleshy or *cauliflower* excrescences attached to all of them. Both the ventricles were quite filled with a dense, white coagulum, firmly attached to their parietes. The commencement of the aorta appeared unusually narrow, in proportion to the heart and to the subject; that of the pulmonary artery greatly enlarged. The auricles of the heart were also in a state of considerable dilatation: but the circumstance which attracted attention most was, that the foramen ovale was open; but the manner of this opening must be more fully described. The septum between the auricles exhibited an oval depression, or attenuated space, of about one-third of an inch in diameter, guarded only by a thin membrane; but at one side it was evidently pervious and open, with a rounded and thickened edge. This membrane acted like a curtain or valve; when viewed or pressed from the left auricle, it was closed, the curtain or membrane pressing close, and overlapping the opening. Viewed from the right auricular cavity, or touched with a probe, it opened, and allowed a free passage, fully as large as a goose's quill, compressed so as to exhibit an elongated or oval section. The blowpipe exhibited the same difference of a closed or open space, as it was used from the left or right side of the auricular septem.

There was no trace or appearance of inflammation in the auricular portions of the heart, at least none could be discerned on the inner membrane.

In the case of Spellman, the following explanation might perhaps be hazarded. That previous to his feverish and pulmonic attack, the blood returning from the veins, and meeting no obstacle from entering the pulmonary artery, (which, as already observed, was larger than natural,) passed altogether the narrow auricular aperture, and took the easier and wider opening into the pulmonary artery; the synchronous movements in the left cavities keeping pace with those on the right. The fulness of the left auricle occasioned its contents to close the curtain by pressing from left to right. This exertion was also rendered more powerful by the increased strength of the left ventricle, and the narrowness of the aorta. By this mechanism and procedure, the heart was kept in a condition very nearly approaching a natural state, the cross valve only opening occasionally on some sudden struggle, or mental emotion, which might disturb the pulmonary circulation. But when the bronchial membranes became thickened, the bronchia filled up, and the lungs themselves less pervious from inflammation, an obstacle was presented to the free passage of the blood through the branches of the pulmonary artery, a regurgitation took place, the oval curtain was thrown open by pressure from right to left, and the anomalous circulation now took place to a greater extent, and in a more permanent manner. Additional sources of tumult and irregularity must have arisen from the metastatic inflammation of the heart itself, and the sudden development of the fleshy vegetations which interfered with the proper action of the semilunar aortic valves. Under the pressure of such aggravated sources of suffering, dyspnœa from congested lungs, a perturbed circulation, effusion into the pericardium, the formation of coagula, which nearly filled both ventricles, (all occurring in succession,) the heart ceased to act, and thus the patient's agonies were at an end.*

* Ibid.

*Case of Extirpation of an unusually large Steatomatous Tumor from the Neck.*

The subject of the operation was Michael Legge, aged twenty-seven, residing in the parish of Ballingarry, of a vigorous, athletic constitution, and accustomed to the laborious occupation of a farmer.

About thirteen years ago, after a severe pulling of the right ear, and pressure of the thumb behind the angle of the jaw, one of the lymphatic glands situated there became inflamed, and formed a tumor. It was not, for a few days, attended with much pain, and was entirely neglected during several years, though it increased slowly.

Sixteen months ago, he was examined at a county infirmary, at which period the tumor had not attained half its present size. After a consultation had been held on his case by five professional gentlemen, it was considered not advisable to attempt an operation. Shortly after his return home, while sparring with a fellow labourer, he received a blow on the tumor which produced violent inflammation. Fomentations and warm poultices were applied, which not being productive of any relief, but, on the contrary, being found to increase his sufferings, were of course discontinued.

From this period, and during the space of a year, it grew to double its previous size, and though it had been hitherto but little sensible on pressure, and had produced no inconvenience except occasional shooting pains during changes of the weather, and slight headaches, more alarming symptoms, such as great pain, dyspnœa, and sense of suffocation, ensued, as the tumor grew and protruded into the fauces.

Being about this time on a visit in his neighbourhood, Dr. Daly accidentally met with him, and, after a careful examination of the tumor, was of opinion that its removal might be easily effected.

It occupied nearly the whole of the right side of the face and neck, extending from the zygomatic arch, under which it seemed imbedded, to within two inches of the clavicle, in which direction it measured in its anterior circuit eleven inches and a half.

The ear was pushed up towards the temple, so that its lobe was expanded over the tumor, and the sides of the meatus externus so closely pressed together, that hearing was entirely obstructed on that side.

Posteriorly it extended to the distance of five inches and a half from the lobe of the ear, and anteriorly over the upper and lower maxillæ, and to within an inch of the angle of the mouth: in this direction it measured from behind, round the most projecting point of its surface, to the sulcus in the skin, parallel to the trachea, twelve inches and a half. The skin covering it was in some spots rather of a livid hue, where some superficial ulcers had been produced by the irritating applications formerly used; but, though very tense, it did not adhere to it. The surface was studded with irregular knobs; several veins extended in various directions over the tumor, but the external jugular was found below and behind it, of its natural size. The tumor did not appear to adhere to the maxillæ, as it could be moved a little to either side, and slightly rotated, though it could not be pulled forwards. The carotid was felt beating deeply under it near the clavicle; but, near the angle of the jaw, it could not be ascertained whether the vessel ran through it or under it. The jaws, anteriorly, could only be separated about an inch. On looking into the mouth, the tumor appeared encroaching on both gums; it covered three of the inferior molares, and one of the superior molares had been extracted in order to prevent the tumor from being pinched by it during mastication. The tumor also protruded inwards behind the velum palati, which it pressed forwards and laterally, insomuch that the uvula was completely thrown to the left side, thereby effacing altogether one of the arches of the soft palate. The velum had become more vascular, and, upon the slightest changes of weather, was attacked with inflammation, bringing with it difficult deglutition, and the other painful accompaniments of cynanche tonsillaris. From the sound of the voice, it was evident that this portion of the tumor pressed on one or both of the posterior nares, and the man was latterly obliged to be awakened two or three times at night, as the swelling pressed on the epiglottis, and threatened suffocation. This symptom, together with the tendency to cynanche, and the perfect mobility of the tumor, pointed out at once the propriety and necessity of extirpation, particularly as the patient's state of health was so favorable to it.

The operation was commenced by making two semilunar incisions on a line with the lower jaw, the upper one about an inch below it, and both extended so as to include a portion of integuments fourteen inches in length, by five in breadth, which was left adhering. Upon cutting through the integuments, the platysma myoides, instead of being spread in a sheet over the tumor, was collected at different points into bundles of pale fibres. As Dr. Daly removed these by the knife, and approached the base of the tumor, the latter receded from the face, and assumed a more globular form, with a narrow neck protruding inwards amongst the deep-seated muscles, vessels, &c. in that triangular space formed by the upper and anterior edge of the sterno-mastoid muscle, the base of the under jaw, and the digastric muscle.

Hitherto no arterial branch of any size had been met with, but near the neck of the tumor three large branches were divided, and immediately secured by ligatures: one of them, the posterior auris, which could be felt pulsating under the skin before the operation, another very near it, apparently arising from the facial, and a branch of the lingual, which entered into the base of the tumor, and chiefly supplied its growth. The facial artery remained untouched: we could feel it pushed over near the chin. As the tumor could not be twisted out, the surgeon was obliged to proceed cautiously, raising the layers of fascia with a dissecting forceps, and separating them with the edge and handle of the knife until the parotid gland appeared, the lower lobe of which lay upon the tumor, and was bound down to it by a strong fascia, a part of which Dr. Daly at first cut through, and subsequently so much as to enable him to turn up the lower portion of the parotid. Nearly the whole body of the tumor was now disengaged, with the exception of its neck, which was attached by strong ligamentous bands to the angle and ramus of the jaw on one side, to the mastoid process on the other, above to the parotid gland and its fascia, and to the cartilage of the ear; below, it was more loosely connected, as its adhesions there were torn through with the handle of the knife. Neither the internal nor external carotid, nor jugular vein, was in any way endangered or exposed.

Fortunately a quantity of fatty substance and lymphatic glands, which we cautiously avoided, lay between them and the tumor; and, on dividing the above-mentioned ligamentous bands, the root of the tumor suddenly gave way, the patient exclaiming that his throat was pulled out.

During our previous examinations, as the tumor which we felt and observed in the fauces did not move when the external one was stirred in all directions, the surgeons did not conceive it to be a part of the external tumor, but to be the tonsil and adjoining parts pushed in before it: however, they were deceived in this opinion, as they now found it to be caused by a prolongation of the large tumor. This lesser or internal tumor was of a pyriform shape, the base being in the throat.

The body of the large tumor being in the way, and not wishing to make any incisions in such a deep situation, the surgeons were deliberating for an instant about cutting it off, and then with more ease separating the smaller one; but a slight pull having torn part of the sac which contained the small tumor, (which was much thinner than that which contained the larger one,) a quantity of fatty gelatinous matter gushed out, the tumor contracted, and slipt out of a bed of loose cellular membrane without farther trouble. It was completely removed, not a particle being left behind. The whole tumor weighed two pounds fourteen ounces, exclusive of the portion of its contents which escaped, and which might make one or two ounces more. The blood lost during the operation was chiefly venous, and did not exceed twelve ounces. On looking into the cavity left by the tumor when the parts collapsed, its depth appeared formidable indeed, as the clenched hand might easily be buried behind the jaw, and nothing but the pharyngeal muscles and mucous membrane prevented the fingers from passing down the œsophagus. The epiglottis was easily and distinctly felt, but the tonsil of that side was not to be found: it was supposed to have been either pushed deeply backwards and upwards towards the base of the skull, or, what is more likely, absorbed.

The base of the large tumor lay with its upper edge covering the parotid gland, which was pressed backward, and in great part absorbed; the smaller tumor ran under it, likewise pressing it upwards. The external carotid was pressed inwards, and to one side. The styloid process and its muscles lay below the neck of the tumor, but none of them were laid bare by the knife. The masseter, buccinator, and upper portion of the sterno-mastoid muscle, were felt covered by fatty substance and condensed cellular membrane.

Having carefully sponged the wound, Dr. Daly tied every minute bleeding point which could be perceived, to the number of three, and washed the whole surface of the flaps with a saturated solution of alum. Having saved a sufficient quantity of integuments, the flaps were easily brought into proper position by adhesive plaster. Compresses dipt in cold water were applied externally, and the whole supported by a double-headed roller round the head and neck.

The usual custom of stuffing the wound with dry lint was not adopted, as it was wished to unite as much of it as possible by the first intention, and thereby diminish the subsequent inflammation and suppuration.

The man has been now (July 10th) for some weeks engaged in his usual occupations, the wound having healed completely over the three silk ligatures; the knots and a portion of the threads of which remain within, and produce no inconvenience. The wound measures now scarce a third of its original extent.

Immediately after the operation, the ear of the affected side recovered its position, and the hearing was restored, but he complained of being unable to close the right eyelids; the mouth was slightly turned to the opposite side, but, as the fulness of the cheek of the affected side disappeared, these symptoms considerably improved. However, as several branches of the seventh pair of nerves must have been unavoidably divided during the operation, it is probable that he may always labour under a partial paralysis of the right cheek.*

---

*Case of Indurated Enlargement of the Uterus, successfully treated with Iodine.*

Jane Reilly, aged forty, * * *. After some hesitation, she now acknowledged there was a hard tumor in the vagina, easily felt by herself, and very painful to the touch, which had gradually increased in size, without any discharge from it; and that menstruation had ceased for some months.

Dr. Thetford prescribed various sedatives to assuage pain and procure sleep, and directed the aperients to be continued until the fæcal discharges had become natural.

* Ibid.

This last object being obtained, he commenced an accurate local examination on the 9th of May. The attempt to introduce a finger into the vagina was met immediately within the labia by a projecting substance, which was easily ascertained to be the os uteri, somewhat enlarged, and firmer than natural; beyond, and connected with which, a large tumor of osseous hardness opposed resistance. He succeeded in introducing a portion of the index finger between it and the cavity of the sacrum; but, laterally and anteriorly, any introduction between it and the other bones of the pelvis was impracticable. There could not exist a doubt of the tumor being the uterus of great size, and in a state of induration. Dr. T. ordered alterative doses of the corrosive muriate of mercury, with pills of conium and hyoscyamus, and enjoined a strict attention to the state of the bowels.

This course was persevered in for thirty-six days. Her health amended, and she was able to walk in her room with less difficulty and pain; but neither the size nor hardness of the uterus was reduced, nor was the pain of the pelvic bones much alleviated; and her spirits were quite depressed, from a conviction that her case was incurable.

From the moment that Dr. Thetford ascertained the nature of her disease, he had resolved on prescribing iodine, should the remedies then contemplated fail. Accordingly, on the 14th of June, he directed seven drops of the tincture to be taken three times daily, in a wine-glassful of cold water; which dose was augmented gradually to ten drops, every other medicine being discontinued, except castor-oil when required.

The result was speedily favorable: her spirits revived; appetite greatly improved; intestinal evacuations in general sufficient without any aperient; urine passed in greater quantity at a time, and with diminished pain; ability to take increased exercise; rapidly progressive absorption of the diseased substance of the uterus; periodical return of the catamenia.

On the 2d of August, she was perfectly restored to health, and continues so.

It may be proper to state, that this individual was a patient of St. Thomas's Parish Dispensary, and unable to aid the effects of medicine by requisite diet.*

---

*Case of Poisoning by Cantharides.* By A. W. Ives, M.D. of New York.

On the evening of the 7th of March, 1827, Dr. Ives was called to the House of Refuge for Juvenile Delinquents, to see William Cummings, a healthy athletic lad, seventeen years old. In a paroxysm of anger, he had swallowed about an ounce of Tincture of Cantharides, supposing it to be laudanum. He was seen at nine o'clock, about an hour and a half after the poison was

* Ibid.

taken, labouring under the following symptoms: Hurried respiration, flushed countenance, eyes red and suffused with tears, profuse ptyalism, small but highly accelerated pulse, convulsive agitation and trembling, acute pains in the region of the stomach and bladder, with such exquisite sensibility of these organs that the slightest pressure was instantly followed by general convulsions.

An hour previous to the arrival of Dr. Ives, the apothecary of the house had given the patient a scruple of the Sulph. Zinc, and he had subsequently taken ten grains of the Sulphate of Copper, by the direction of a medical friend. As he had not yet vomited, and the symptoms continued to increase in violence, one drachm of ipecacuanha was administered; and, at the expiration of twenty minutes, he was bled to sixteen ounces from the arm. The operation was followed by faintness and free vomiting, and the convulsive action became for awhile suspended. He was now ordered two ounces Ol. Ricini, and, to accelerate its operation, frequent mucilaginous injections; and, should these fail of producing free evacuations, the oil to be repeated in four hours. He was also directed to drink copiously of linseed tea, to have fomentations applied to the abdomen, and, in case of a recurrence of the convulsions, a laudanum injection after the operation of the cathartic.

It was found necessary to repeat the oil in the course of the night, after which full dejections took place. Convulsions of the limbs occurred at intervals, and also long-continued and painful priapisms.

On the morning of the 8th, the patient was in some degree relieved from the violent symptoms of the preceding evening. His mind was calm; there was but little febrile excitement, though his pulse was quicker and skin somewhat dryer than in health. He was still unable to bear the least pressure over the epigastric and pubic regions, and he had passed no urine.

He was directed to be cupped over the hypogastrium; to take of the following mixture: Sp. Eth. Nitric. ℥ss.; Sp. Acet. Ammon. ℥iv. M. half an ounce every two hours.—Linseed and barley tea at four o'clock P.M.—Repeat the Ol. Ricini, fomentations, pediluvium.

9th, nine o'clock A.M.—During the preceding day he continued comfortable; full evacuations followed the use of the oil, and the spasms disappeared. In the evening, however, an exacerbation of the symptoms ensued; the pain about the bladder increased; priapism returned, and, before the next morning, wild delirium. At this hour he is composed, complains of tenderness over the bladder, particularly on pressure, but is calm and comparatively comfortable; pulse eighty in a minute, with slight increase of action; a thin white coat upon the tongue.

V.S. to ℥viij.—Ol. Ricini at two o'clock P.M.—Fomentations to be continued through the day.

10th.—At eight o'clock this morning, Dr. Ives found him in a state of complete insensibility. About seven o'clock, the evening before, the convulsions returned with appalling violence, and continued for two or three hours, when there was an interval of quiet and reason. At two o'clock in the morning, after having complained for a short time of severe pain in the head, he sunk into a state of coma, in which he continued at the time of the visit.

Semicupium. Strong sinapisms to the back of the neck, feet, ankles, and legs. Warm stimulating injections.

11th.—At ten o'clock A.M. the condition of the patient apparently much improved. Four hours after the last visit, he had a copious evacuation from the bowels, and at the same time voided eight or ten ounces of high-coloured urine. In the course of the day he occasionally complained of pain in the head, and at times had slight delirium, but these symptoms subsided after repeating the sinapisms. He has passed no water since yesterday afternoon.

Blister to the nape of the neck. Sinapisms above the pubes.—Calomel ten grains, to be followed by Castor-oil.

The evacuations from the bowels were from this period free and natural, and the healthy secretion of the urine was restored. The patient recovered rapidly, so that in one week he was able to return to his customary employment, and to eat with a good appetite of the usual diet of the house. He seemed to regret sincerely the folly and rashness that had well nigh destroyed him; and from this time was obedient, faithful, and industrious, and apparently free from all symptoms of disease, until the 14th of April.

About ten o'clock this day, whilst employed in whitewashing, he was suddenly seized with severe pain in the head, pain in the right side, chilliness, and trembling, and shortly afterwards with universal spasms. (He was from this time under the immediate direction of Dr. Stearns, who was in attendance at the Refuge, and in the hospital, when Cummings was brought in.) He was immediately directed an emetico-cathartic, and during the reaction which succeeded the attack was bled largely.

By these means he appeared to be relieved of pain, and the spasms subsided; but he soon after sunk into a comatose state, and remained in it till the next morning.

Fomentations. Pediluvium. Sinapisms to the thighs and feet. Blister to the nape of the neck.

15th.—In addition to the above means, he was this morning directed fifteen grains of Calomel, and subsequently Sulphat. Soda in sufficient quantity to promote free evacuations from the bowels. He was again for a few hours roused from lethargy, but at one o'clock P.M. relapsed; and thus, as the force of disease or the power of remedies predominated, was alternately comatose and rational till morning.

16th.—He spoke rationally and intelligibly, and his pulse, eyes, skin, and general appearance, seemed to indicate a favorable crisis. He was now put upon the use of small doses of Calomel and Dover's powder, as an alterative.

At five o'clock of the same day, he was again attacked with severe convulsions, which continued about four hours.

17th.—Better. A slight return of spasms in the afternoon.

18th.—His mouth and gums are affected by the calomel. From this time he appeared to be rapidly improving till the 20th, when he had so far recovered his strength as to sit up the whole day. The only unfavorable symptom observable at this time was an unalterable conviction in his own mind that he should shortly die.

21st.—At four o'clock P.M. he had a recurrence of terrible convulsions, which lasted about half an hour; then a lucid interval of four or five hours; and finally a state of entire insensibility, which continued till ten o'clock A.M. of the 22d, when he expired.

At four o'clock of the same day the body was opened, and, by the assistance of Drs. WOOD, CHEESEMAN, and RODGERS, the contents of the cranium, thorax, abdomen, and pelvis, were examined with unusual particularity.

The only morbid appearances exhibited were the following: The blood-vessels of the brain were preternaturally turgid, especially those of the cerebellum, which was covered with a coat of coagulable lymph. About an ounce of serum was effused into the base of the skull. The mucous membrane of the stomach was whitish, or paler than natural, soft, pulpy, and very easily detached by rubbing it with the finger. Sanguineous congestion in the pelvis of the kidneys, exhibiting the usual appearances of inflammation in these parts. Ten ounces of urine were drawn from the bladder by a catheter.*

* American Journal of Medical Sciences.

