# Errata

**Published:** 1828-08

**Authors:** 


					P. 50, 1. 29, for views read veins.

				

